# Setup of Galvanic Sensors for the Monitoring of Gilded Bronzes

**DOI:** 10.3390/s140407066

**Published:** 2014-04-22

**Authors:** Sara Goidanich, Davide Gulotta, Laura Brambilla, Ruben Beltrami, Paola Fermo, Lucia Toniolo

**Affiliations:** 1 Department of Chemistry, Materials and Chemical Engineering “Giulio Natta”, Politecnico di Milano, Via Mancinelli 7, 20131 Milano, Italy; E-Mails: davide.gulotta@polimi.it (D.G.); lucia.toniolo@polimi.it (L.T.); 2 Haute-Ecole Arc Conservation-Restauration, Espace de l'Europe 11, 2000 Neuchâtel, Switzerland; E-Mail: laura.brambilla@he-arc.ch; 3 Chemistry Department, University of Fribourg, Chemin du Musée 9, CH-1700 Fribourg, Switzerland; E-Mail: ruben.beltrami@unifr.ch; 4 Department of Chemistry, University of Milan, Via Golgi 19, 20133 Milan, Italy; E-Mail: paola.fermo@unimi.it

**Keywords:** galvanic sensors, gilded bronze, *in situ* monitoring, corrosion, conservation

## Abstract

Traditional electrochemical techniques, such as linear polarization resistance (Rp), and electrochemical impedance spectroscopy (EIS), cannot be applied to gilded bronzes, as it may not be possible to interpret the results obtained due to the bimetallic nature of the studied material. The measurement of the macrocouple current generated by the gold/bronze galvanic couple can be used as an indicator of degradation processes. Nevertheless, this measurement cannot be performed directly on the original artifacts due to the systematic presence of short-circuits between the two metals. In the present work the use of galvanic sensors is proposed as a possible solution for the monitoring of gilded bronze artefacts. The sensors have been designed to simulate real gilded bronze surfaces in terms of composition and stratigraphy and have proved to be a reliable diagnostic tool for the *in situ* monitoring of the rates of deterioration of gilded bronze surfaces and to test new conservation treatments. Their set-up and application is reported and their performances discussed.

## 1. Introduction

Non-destructive and *in situ* monitoring is a fundamental diagnostic approach supporting the preservation strategies of both museum collections and cultural heritage artifacts exposed outdoors. It is also of utmost importance when new conservation strategies or treatments are developed and tested [1]. The preservation of metallic surfaces, in particular, can take advantage of some consolidated electrochemical techniques [2–4], such as potential measurements, linear polarisation resistance (Rp) and electrochemical impedance spectroscopy (EIS). Such techniques have been adapted for *in situ* application by using contact-probes, and can therefore provide important information about the state of conservation of objects [5–19]. Recently, some European research projects [20–25] have been aimed to the development of innovative tools to assess the risk of corrosion of cultural heritage artifacts and to improve the preventive conservation policies. In particular, as far as indoor conservation is concerned, conditions of “low corrosivity” of museum atmospheres and the occurrence of even slight environmental variations are key factors to be monitored in order to predict the future evolution of corrosion rates. The electrical resistance (ER) technique [20–26] has proved to be a very efficient tool for such applications in archives, libraries and museums. Coupons and sensors are exposed to low-corrosive museum atmospheres, periodically removed and analyzed. The results provide quantitative information on the corrosion rate of the reference materials. Moreover, the characterization of the corrosion products formed over the coupons allows the identification of the pollutants responsible for the damage.

However, none of the previously mentioned techniques can be applied to the monitoring of gilded bronzes, due to the bimetallic nature of such objects and to the complexity of the resulting data (which can be hard to interpret). It is worth noting that the conservation of gilded bronzes often represents a critical issue in the field of cultural heritage. The exposure to pollution and adverse environmental conditions promote the formation of unstable corrosion products at the gold/bronze interface, which can hardly be removed without damaging the gilding. Moreover, the reactivity of the corrosion products in the presence of some of the most common atmospheric pollutants, namely nitrates and sulfates, promotes the formation of further less stable compounds, which occurs with volume variations. As a result, the related mechanical stress at the gilding interface promotes bursting effects, induces progressive loss of adherence, and can ultimately result in the detachment of the gold layer. The growth of crystals of unstable corrosion products can also deteriorate the overlaying gilding causing cracks and surface discontinuities. Corrosion evolution and chemical transformations of the patina occur at an appreciable rate only if liquid water is available (generally resulting from surface condensation) [27]. Water condensation takes place on a solid surface through several mechanisms. Among all, chemical condensation is particularly dangerous because it takes places at rather low relative humidity values (RH). The presence of hygroscopic salts, such as chlorides or ammonium compounds, favors water absorption and increases the conductivity of patinas, thus enhancing the electrode reactions. In addition, galvanic coupling between gold and bronze further accelerate the rate of corrosion of the underlying bronze. Due to the potential harmfulness of the previously discussed damaging factors, unstable cultural heritage gilded bronzes are often removed from their original locations and stored in museums under controlled conditions in order to assure their preservation [28–31]. It is therefore of great importance to be able to monitor such precious and delicate objects. The bimetallic nature of gilded bronzes suggests the possibility of monitoring the macrocouple current flowing between bronze and gold, which is directly correlated with the corrosion rate. The occurrence of unavoidable short-circuits between the two metals does not allow direct measurements on the original artifacts. The use of galvanic sensors (which allow the monitoring of the macrocouple current exchanged between two different metals), specifically designed to simulate the actual gilded bronze artifacts, can provide a valid alternative. In order to avoid short-circuits and therefore to allow the macrocouple current measurements it is necessary to have a patina of corrosion products separating the gilding from the metal substrate.

The realization of artificial patinas with specific and reproducible characteristics (thickness, porosity, cohesion and composition) it is therefore a crucial point for the setup of suitable galvanic sensors. Patinas should also be representative of the naturally-formed ones on real bronze objects. The most common constituents of natural patinas are copper oxide (cuprite), basic copper sulfates, such as brochantite or antlerite, together with copper chlorides [27,32–40]. In marine exposure conditions, copper chlorides, such as atacamite or nantokite, are the main corrosion products. Nantokite, when present, can turn into basic copper chlorides, such as atacamite, as a result of the interaction with atmospheric moisture. Such a transformation is one of the main causes of a the so-called “bronze disease” [32,41]. A number of publications provide indications to prepare natural and artificial patinas in the laboratory [42–47], but for some of the most important ones (such as those containing brochantite), good reproducibility can only be obtained after highly time-consuming ageing procedures.

**Figure 1.**
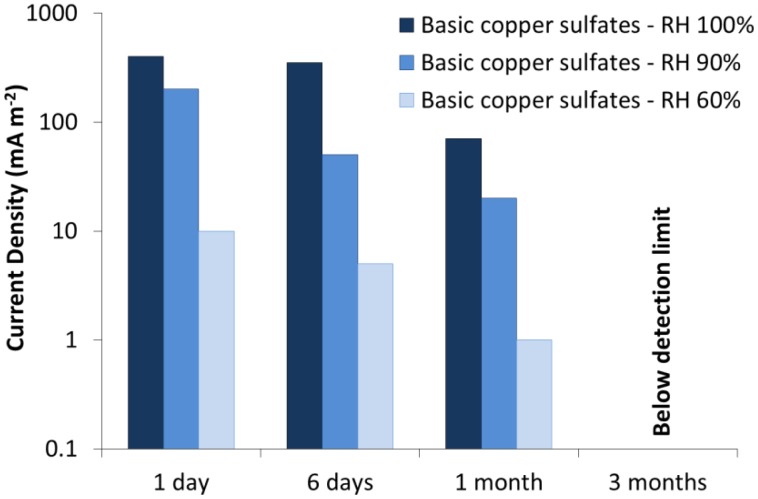
The galvanic sensors of the seventies [48,49]: macrocouple current as a function of time and relative humidity.

The first galvanic sensors simulating gilded bronzes were designed during the seventies to evaluate new conservation strategies for Lorenzo Ghiberti's *Porta del Paradiso* [48,49]. At that time the possibility to maintain the *Porta del Paradiso* in its original location was under discussion and the first sensors were therefore designed to operate in outdoor conditions under high relative humidity values: up to 100% RH with the presence of superficial moisture [48–50]. The metallic substrate of the sensors was a quaternary bronze (83.90% Cu, 5.80% Sn, 4.25% Pb, 5.30% Zn, 0.58% Ni), and the artificial patina of corrosion products was mainly constituted by basic copper sulfates. The gilding was obtained by applying a thin gold sheet glued along the lateral edges of the sensor. The maximum current density was reached during the first 24 h and for 100% RH (Figure 1). The current density then decreased quickly with time, falling below the detection limit after three months. Those sensors therefore could only be used to monitor gilded bronzes for a short time period and in quite humid conditions (RH ≥ 60%).

Nowadays, the main request arising from conservators and museum curators is the long-term monitoring and microclimate control for the preventive conservation and maintenance of restored gilded bronzes, which often still remain in quite unstable conditions [28–31]. New galvanic sensors are thus needed to provide quantitative corrosion rate data even under very low environmental corrosivity conditions. A preliminary version of such sensors has been designed to identify the most suitable environmental conditions for the long-term display of the *Porta del Paradiso* [30] at the *Museo dell'Opera del Duomo* of Florence. In this case, the sensors provided important monitoring data even under very low corrosivity conditions (nitrogen atmosphere and very low RH). Nevertheless, they demonstrated low durability and poor reproducibility of the acquired signals.

In the present paper the setup of new galvanic sensors is described. Their characteristics and monitoring performances are analyzed and compared with the previous versions. In particular, significant modifications of the methodology for the realization of the artificial patina and of the gilding procedure have been made in order to improve both durability and reproducibility. The effect of such variations on the sensors characteristics is also discussed.

## 2. Experimental Section

### 2.1. Galvanic Sensors

**Figure 2.**
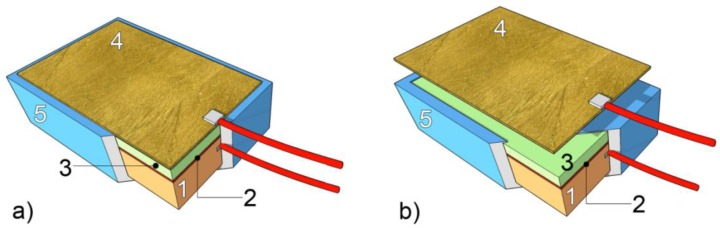
General model of a galvanic sensor (**a**) with detail of the gold leaf overlapped (**b**).The complete stratigraphy of the sensor includes: (1) metallic substrate; (2) cuprite layer; (3) patina of corrosion products; (4) gilding; (5) electric insulation cover.

The scheme and a picture of the galvanic sensors setup used in the present study are given in Figure 2.

Pure copper (Cu 99.90%) and a quaternary bronze alloy, (Cu 93,1%; Zn 3,2%; Sn 2,6%; Pb 1,1%) have been used as metallic substrates for the realization of the galvanic sensors. The stratigraphy of the sensors can be described as follows: (1) metallic substrate; (2) cuprite layer (Cu_2_O); (3) artificial patina of corrosion products; and (4) gilding.

**Figure 3.**
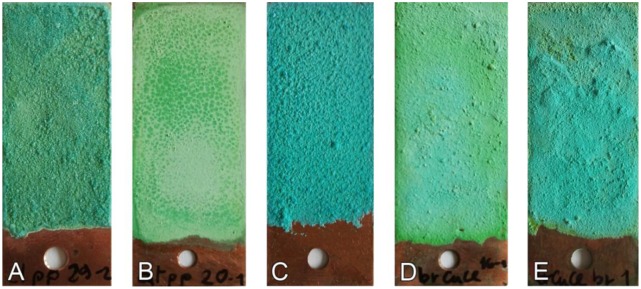
Photographic documentation of the different types of patina: S&C (**A**); BCC (**B**); BrocChal (**C**); BrocNan (**D**); BrocNan+Broc (**E**) (specimens dimensions: 60 × 20 mm).

Prior to the preparation of the sensors, the copper substrates have just been degreased in acetone, while the quaternary bronze substrates were milled in order to remove all the undesired corrosion products. This way, a homogeneous surface has been achieved. The cuprite layer is obtained after a 30–60 min immersion of the metallic substrate in a boiling solution containing: (a) 25 g/L copper sulfate for bronze, and (b) 6.5 g/L copper sulfate, 1.25 g/L copper acetate, 2 g/L sodium chloride and 1.25 g/L potassium nitrate for copper [45]. The cuprite layer could enhance the adhesion of the corrosion products to the sensor substrate and its efficiency is currently under evaluation. This layer should therefore be considered as optional with respect to the overall stratigraphy. Tests have been conducted on sensors with and without the cuprite layer; however the results presented here always refer to a complete stratigraphy, thus including the cuprite layer. The artificial patina of corrosion products has been prepared according to the “applied paste” method described by Hughes and Rowe [45]: copper compounds (selected according to the type of patina, see details in Table 1) are finely grounded in a mortar; water is added until a paste with adequate fluidity is obtained; the paste is then finally spread on the metallic surface by means of a stainless steel spatula. The “applied paste” method allows the preparation of artificial patinas with a wide range of different compositions, which are representative of the various conservation conditions of ancient gilded bronzes. Moreover, the thickness of the artificial patinas can be tuned by overlapping additional layers (up to three overlapped layers have been tested), having either a similar or a different mutual composition. The thickness has been verified by using a micrometric gauge. The characteristics and recipes of the artificial patinas which have been set up for the preparation of the galvanic sensor are summarized in Table 1. The types of patina to be reproduced and tested in the present study have been selected according to their relevance in the conservation of gilded bronze artifacts, as reported in the scientific literature [28,29,31,51–53]. All patinas have been used for the preparation of both copper and bronze sensors. Photographic documentation of the obtained patinas is shown in Figure 3.

The gilding is obtained by applying a gold leaf on the artificial patina, before it gets completely dry. The gold leaves used in this work are composed by gold 24 KT (999.9/1,000), 80 × 80 mm and thickness 20. The value of the thickness provided by the supplier refers to the grams used to produce 1,000 leaves of the same dimensions. The procedure to obtain the gold leaf is still manual and so the thickness of each leaf can vary. The average thickness of the gold leaf (0.16 μm) has been calculated accordingly and confirmed by SEM observation. The humid conditions of the patina during the gilding operation provide an adequate adhesion to the gold leaf so that no additional adhesive is necessary. Several drying times at a constant relative humidity of 35%–40% (RH%) have been tested in order to identify the best substrate conditions for gilding. The optimal drying times required for each patina composition prior to gold leaf application are reported in Table 2.

Each sensor has electrical connections both to the metallic substrate and to the gilding for the measurement of the macrocouple current: the connection to the substrate is obtained by a metal rivet, while the gold leaf is connected by means of conductive silver-based glue applied on its external surface. The lateral sides, the back and all the connections of the sensor are coated with epoxy resin in order to ensure the electrical insulation of the entire system.

### 2.2. Test Equipment

The macrocouple current flowing between the gilding and the bronze is directly correlated by Faraday's Law, to the corrosion rate and is continuously monitored by means of a high precision Keithley 3706 multimeter. The same equipment has been used to measure the driving force for the galvanic corrosion and the electrical resistance of the artificial patina. The resolution of the multimeter is 0.01–100 μV in the 100 mV–300 V range, 0.1 μΩ–10 Ω in the 1 Ω–100 MΩ range and 1 pA–1 μA in the 10 μA–3 A range.

Stereomicroscopic observation of the patina and of the sensors surface has been performed using a Leica M205C stereomicroscope, equipped with a Leica DFC290 digital camera.

Infrared spectra of patinas were recorded in transmission mode, in the spectral range between 4,000 and 400 cm^−1^ with 4 cm^−1^ resolution, using a Thermo Electron Nicolet 6700 FTIR spectrometer.

A Philips PW1830X-Ray Diffractometer (XRD) with a PW3020 generator, in the Bragg-Brentano geometry and Thin Film, copper anticathode (Kα1 radiation;, was used for phase identification of patinas.

Environmental Scanning Electron Microscope (ESEM) was performed using a Zeiss EVO 50 EP instrument equipped with a LaB6 source and an Oxford INCA 200—Pentafet LZ4 X-ray spectrometer was used to observe patina morphology and to determine elemental compositions.

### 2.3. Chemicals

Nantokite (copper(I)chloride, ACS reagent, ≥ 90%), eriochalcite (copper(II)chloride dihydrate, reagent grade) and chalcantite (copper(II)sulfate pentahydrate, BioReagent ≥ 98%) are Sigma Aldrich products. The basic copper chlorides (Cu_2_(OH)_3_Cl) and brocantite powder have been synthesized in the laboratory. Basic copper chlorides are obtained just by leaving some nantokite powder at RH > 80% for 72 h. Then it has to dry at 60 °C for 24 h. By XRD it is verified that the conversion into basic copper chlorides is complete and that the residual nantokite is very low.

Brochantite is synthesized in the laboratory following a procedure reported by Prasad and coauthors [54]. Silver glue was provided by Agar Scientific.

### 2.4. Exposure Conditions

Galvanic sensors have been kept in a climatic chamber in the laboratory. A constant temperature of 25 ± 3 °C has been maintained for all the time. The relative humidity has been periodically varied ranging from 5% to 75%. Temperature and relative humidity have been monitored by means of ELR200 LSI Lastem T-RH sensors, −20/60 °C T range, 0/100% RH range, ±0.5 °C T accuracy, ±2% RH accuracy.

## 3. Results and Discussion

### 3.1. The Artificial Patina

The artificial patina is a key factor for the realization of galvanic sensors with good durability and sensitivity, even to small environmental variations, under very low-corrosivity condition (e.g., RH% below 20%). Thickness and adhesion to the substrate are the two most important factors. Several chemical and electrochemical patination methodologies have been preliminary tested with unsatisfactorily results [55]. These methods have been discarded being whether highly time-consuming or due to the lack of reproducibility of the expected final characteristics of the galvanic sensors. The “applied paste” method previously described has been therefore selected and set up.

**Figure 4.**
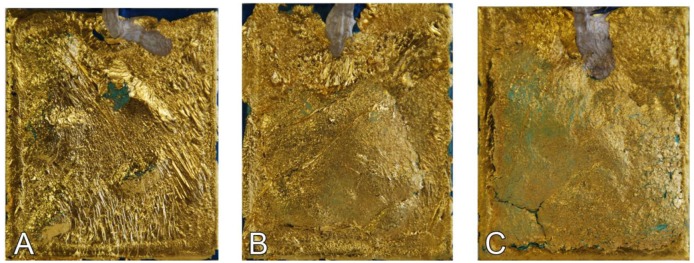
Photographic documentation of galvanic sensors with increasing thickness of the patina realized on copper substrate and with a S&C patina: single layer (**A**), double layer (**B**) and triple layer (**C**) (sensors dimensions: 60 × 50 mm).

On the one hand, the patina should be thick enough to avoid short circuits between the gilding and the metallic substrate. On the other hand as the thickness of the patina increases, the overall durability of the sensors tends to be reduced. This phenomenon is mainly associated to the presence of hygroscopic compounds (e.g., chlorides) within the patina, that tend to blister and to detach from the metallic substrate as a result of the volume change triggered by relative humidity variations. As it can be observed in Figure 4, after few months under laboratory conditions and variable RH (10%–70%), the S&C single layer shows limited blistering and gilding detachment and is much more stable and durable compared to the double and triple layer. The latter sensor, in particular, completely degrades showing an almost complete detachment of the artificial patina.

During the early stages of the study, an intermediate cuprite layer was introduced between the metal substrate and the artificial patina, in order to improve the adhesion of the latter [55]. No evidence of such an effect has been demonstrated so far, whereas recent data have shown that a high surface roughness of the substrates seems to favor the adhesion of the patina. Further tests are currently in progress in order to better understand the real influence of both factors with respect to adhesion.

**Figure 5.**
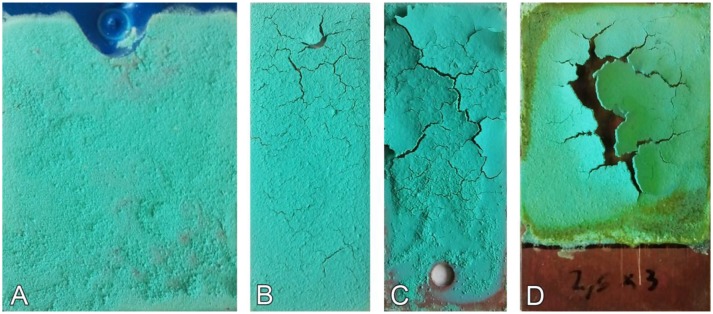
Brochantite patinas with increasing thickness (**A**) > (**B**) > (**C**) > (**D**) (sensors max. length: 60 mm).

The composition of the patina can be changed in order to reproduce as close as possible the actual stratigraphy of the original object to be monitored or to be treated with new conservative procedures. Unfortunately, it has not been possible to realize in a reproducible way pure brochantite sensors, which simulate the most diffuse corrosion product on copper alloys artifacts in urban environment. Brochantite patinas are generally characterized by a very low internal cohesion, a limited adhesion to the substrate and a high tendency to form cracks (Figure 5). In particular, as previously discussed, the number of cracks increases as the thickness of the patina grows. The only way to obtain a suitable brochantite patina is to apply a very thin paste (Figure 5A), which however shows scarce cohesion and a rather powder-like aspect. Moreover, such a patina has a very high risk of short circuits between the gold and the metallic substrate.

Patina composition and morphology have been characterized using different analytical techniques, such as FTIR, XRD and SEM-EDX (data not shown).

### 3.2. The Gilding

Gilding is another crucial step for the realization of galvanic sensors. The aim is to obtain a resistant and homogeneous gilding without any glue that could reduce the macrocouple current and would introduce an incompatible material with respect to the gilded bronze system to be simulated (as occurred in the sensors of the seventies).

The gilding was initially obtained by sputtering [30]. This procedure has since been discarded due to the fragility of the obtained gold layer, which significantly reduced the durability of the sensors. Gold leaf gilding has been then tested and set up. This procedure has provided much thicker and durable gildings compared to the sputtered ones.

**Figure 6.**
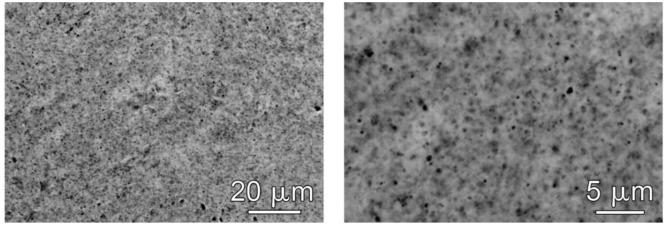
SEM documentation of the characteristic porosity of the gold leaf.

**Figure 7.**
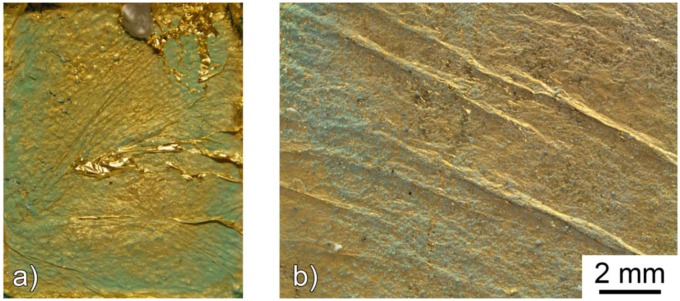
Macroscopic (sensor dimensions: 60 × 50 mm) (**a**) and microscopic (**b**) documentation of a galvanic sensors resulting from the too early application of the gold leaf on a highly moistened patina.

The timing for the application of the gilding is the key factor of this methodology. As previously discussed, the humid nature of the patina enhances the adherence to the metal substrates. Nevertheless, a too high moisture content of the patina can promote migration of soluble salts through the porosity of the gold leaf, thus causing surface crystallization. In Figure 6 the characteristic porosity of the gold leaf is reported. As it can be observed, the porosity is not homogeneous and the maximum pore diameter is generally below 1 μm. Figure 7 shows the typical aspect of a sensor in which the gold leaf has been applied too early on a very humid patina.

**Figure 8.**
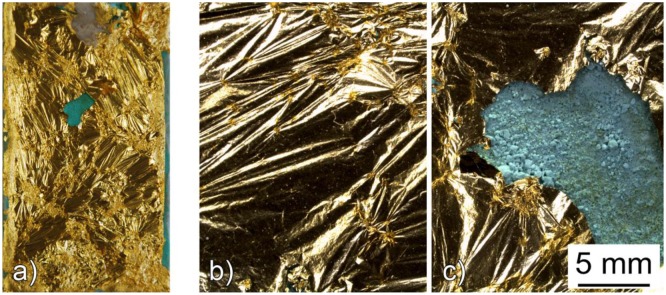
Macroscopic (sensors max. length: 60 mm) (**a**) and microscopic documentation (**b**,**c**) of a galvanic sensors showing a lack of adhesion of the gold leaf.

On the other hand, the application of the gold leaf on a too dry patina will determine a limited adhesion to the substrate, as shown in Figure 8.

### 3.3. The Galvanic Sensors

**Figure 9.**
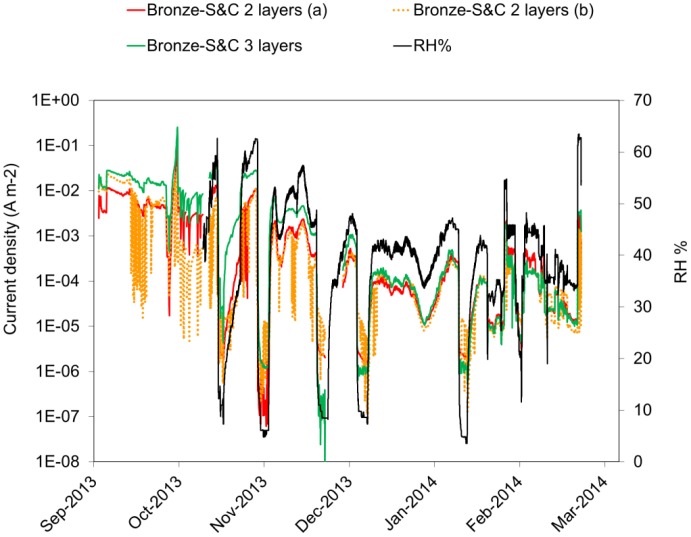
Macrocouple current as a function of time for three galvanic sensors with bronze substrate and double or triple layer of S&C artificial patina.

**Figure 10.**
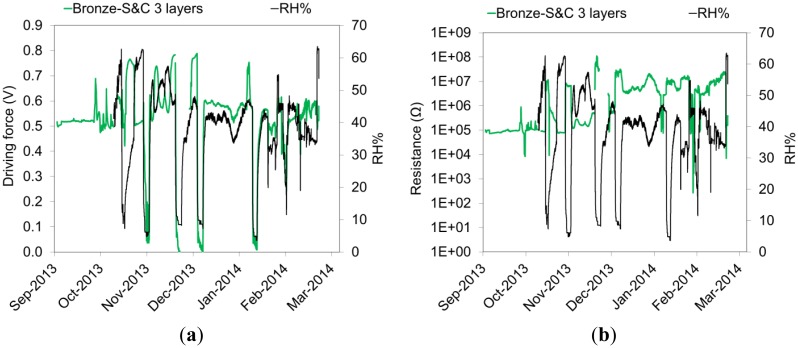
Driving force (**a**) and Resistance (**b**) as a function of time a galvanic sensors with bronze substrate and triple layer of S&C artificial patina.

Galvanic sensors allow a continuous monitoring of the macrocouple current flowing between the gilding and the metallic substrate (Figure 9), of the driving force for the galvanic corrosion (Figure 10a) and of the electrical resistance of the artificial patina of corrosion products (Figure 10b). Data show that all these parameters are strongly linked to the variation of the relative humidity. Corrosion products such as copper chlorides are highly hygroscopic and tend to absorb moisture from the air when the RH rises. As a result, patinas become more conductive and the galvanic corrosion is enhanced. They also confirm that during the conditioning time the driving force and resistance trends are still affected by the initially high moisture content of the patina. The monitoring of these parameters therefore allows the evaluation of the effects induced by the environmental variations on the rate of corrosion of gilded bronzes. In particular, the macrocouple current is directly correlated by the Faraday's law to the corrosion rate. In this paper only macrocouple current data are reported and discussed, so that the assumptions required for the conversion from current density to corrosion rate (e.g., μm·y^−1^, g·m^−2^·y^−1^) are avoided. These assumptions include: (a) corrosion is generalized; (b) if alloys are considered, the corroded elements and the involved anodic reactions have to be individuated; and (c) the effective cathodic and anodic areas have to be calculated, also taking into account the surface roughness. As it can be observed in Figure 9, galvanic sensors with the same composition of the artificial patina are characterized by a quite reproducible macrocouple current. This result confirms that the reproducibility of the galvanic sensors with the new setup has significantly improved compared to the previous version used for the study of the exposition conditions of the *Porta del Paradiso* [30].

**Figure 11.**
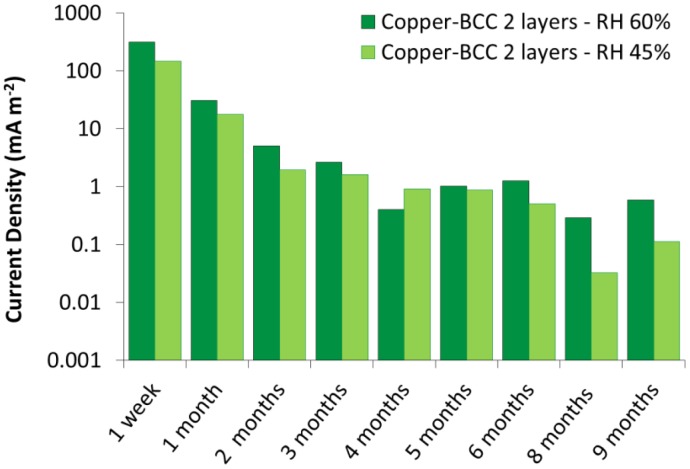
Average macrocouple current density detected at constant temperature (25 °C) and relative humidity (60% and 45%) for galvanic sensors with copper substrate and double layer of BCC artificial patina.

The galvanic sensors require a certain conditioning time in order to reach stability. This is mostly related to the artificial patination procedure which produces highly moistened corrosion layers. As a consequence, the macrocouple current, and therefore the corrosion rate, is generally higher during the early stage of sensor exposition, as it can be observed in Figure 11, where the average macrocouple current density detected at constant temperature (25 °C) and relative humidity (60% and 45%) is reported. The values refer to a couple of galvanic sensors with copper substrate and double layer of BCC artificial patina. In general a conditioning time of approximately two months appears to be sufficient for all the galvanic sensors realized so far. The conditioning time increases when the artificial patina is thicker. As it can be observed in Figure 9, for the first two months, the three layers sensor presents a higher macrocouple current density compared with the two double layer galvanic sensors, which can be ascribed to its initially higher water content. As the moisture evaporation proceeds, the three sensors show a very similar macrocouple current.

**Figure 12.**
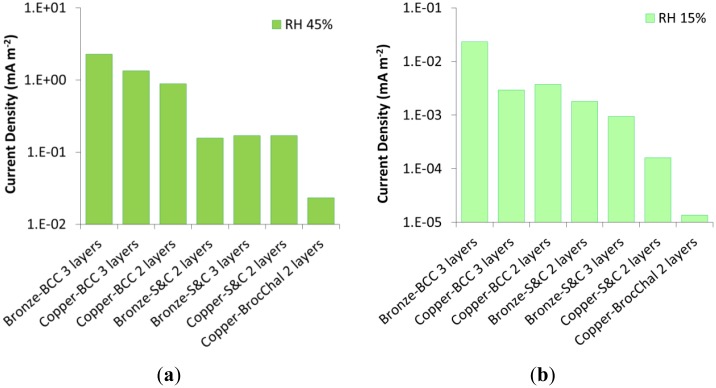
Average macrocouple current density as a function of the artificial patina composition and thickness, detected at constant temperature (25°C) during the fifth month of life of the galvanic sensors at RH% 45% (**a**) and 15% (**b**).

As it can be observed in Figure 12, there are no significant differences in terms of macrocouple current between sensors realized on copper or bronze substrate. The slightly higher macrocouple current density observed for the bronze substrates has been mainly ascribed to their rougher surface before the application of the artificial patina that could have guaranteed a better adhesion. This aspect requires further investigation.

The composition of the artificial patina plays a very important role. Variations of almost one order of magnitude have been observed as a result of different compositions of the patina; being the basic copper chlorides the ones with the highest corrosion rates. Therefore, the design of the most suitable galvanic sensors for the monitoring of a specific gilded bronze artifact must rely on a thorough knowledge of the real composition and stratigraphy of the corrosion layers.

The macrocouple current can be detected even at very low relative humidity (15%). These sensors can therefore be used to study the behavior of gilded bronzes even in the case of very low environmental aggressiveness.

So far, the galvanic sensors have been continuously monitored for nine months and they demonstrate a quite stable and reliable behavior. However, those containing chlorides within the artificial patina show some blistering and some cracks as a consequence of the several cycles of high and low humidity. This is associated to the progressive but very slow reduction of the macrocouple current, while sensors containing only copper sulfates show no sign of degradation and proved to be very durable.

One of the advantages of the galvanic sensors compared to other electrochemical techniques, such as Rp and EIS, is that their surface does not require any moistening to perform the measurements. Data can be therefore acquired under the same environmental conditions under which the artifacts are maintained, while Rp and EIS reflect the behavior of a continuously-wet surface which hardly ever occurs in real museum conditions. It has to be pointed out that Rp and EIS can be performed directly on the objects while the galvanic sensors are meant to simulate the original surfaces. The sensors are therefore an important tool for the definition of the conservation strategies, as they can be used as “replica” of the original artifacts, in order to test new treatments or to study the degradation mechanisms.

## 4. Conclusions

Galvanic sensors for the study and the monitoring of gilded bronzes exposed in indoor conditions have been developed and carefully described. The new setup of the galvanic sensors guarantees a higher durability, reproducibility and reliability compared to all the previous ones. In particular, they proved to be extremely sensitive in the monitoring of the corrosion rate even in low corrosivity conditions.

An important characteristic of galvanic sensors is that corrosion rates can be monitored under the same environmental condition of the artifacts and that they can be used as “replicas” of the original objects. The realization of the artificial patina and the application of the gold leaf are two key steps influencing the performance of the sensors.

A set of galvanic sensors is currently under study for the definition of the optimal environmental parameters for the preservation of gilded bronzes in different conservation conditions (e.g., composition of corrosion layers). A further set with different patina compositions is now installed behind the *Porta del Paradiso* at the *Museo dell'opera del Duomo* of Florence and contribute to the monitoring of this Renaissance masterpiece.

## Figures and Tables

**Table 1.** List and preparation features of the artificial patinas.

**Name**	**Ingredients**	**Powder per Each Layer (g/cm^2^)**	**W/P (Water to Powder Ratio)**	**Composition of the Artificial Patina**	**Number of Applied Layers**	**Thickness (mm)**
*S&C (Sulfates and Chlorides)*	Chalcantite (CuSO_4_·5H_2_O)	0.025	1	Chalcantite (CuSO_4_·5H_2_O)	1	0.6
	Nantokite (CuCl)			Nantokite (CuCl)	2	0.9
	Eriochalcite (CuCl_2_·2H_2_O) (Ratio 4:3:1)			Atacamite (Cu_2_(OH)_3_Cl)	3	1.1

*BCC (Basic Copper Chlorides)*	Mainly basic copper chlorides (Cu_2_(OH)_3_Cl) with low amounts of residual copper chlorides	0.010	2	Mainly atacamite (Cu_2_(OH)_3_Cl) with low amount of residual copper chlorides	1	0.5
					2	0.6
					3	0.7

*BrocChal*	Brochantite (Cu_4_(SO_4_)(OH)_6_) Chalcantite (CuSO_4_·5H_2_O) (ratio 1:1)	0.025	1	Brochantite (Cu_4_(SO_4_)(OH)_6_) Chalcantite (CuSO_4_·5H_2_O)	1	0.8

*BrocNan*	Brochantite (Cu_4_(SO_4_)(OH)_6_) Nantokite (CuCl) (ratio 1:1)	0.025	1	Brochantite (Cu_4_(SO_4_)(OH)_6_) Nantokite (CuCl) Atacamite (Cu_2_(OH)_3_Cl)	1	0.7
					2	0.9

*BrocNan* + *Broc*	First Layer: Brochantite and nantokite (1:1)	1st: 0.025	1st: 1	Brochantite (Cu_4_(SO_4_)(OH)_6_)	2	1.0
	Second Layer: Brochantite	2nd: 0.007	2nd: 3.4	Nantokite (CuCl) Atacamite (Cu_2_(OH)_3_Cl)		

**Table 2.** Drying time of the artificial patina at 35%–40% RH before the application of the gold leaf.

**Name**	**Drying Time before Gold Leaf Application (min)**
*S&C*	30
*Atac*	45
*BrocChal*	30
*BrocNan*	40
*BrocNan*+*Broc*	35
